# Characterization of Bone Marrow Mononuclear Cells on Biomaterials for Bone Tissue Engineering *In Vitro*

**DOI:** 10.1155/2015/762407

**Published:** 2015-02-23

**Authors:** Dirk Henrich, René Verboket, Alexander Schaible, Kerstin Kontradowitz, Elsie Oppermann, Jan C. Brune, Christoph Nau, Simon Meier, Halvard Bonig, Ingo Marzi, Caroline Seebach

**Affiliations:** ^1^Department of Trauma, Hand and Reconstructive Surgery, Hospital of the Goethe University, Theodor-Stern-Kai 7, 60590 Frankfurt am Main, Germany; ^2^Department of General and Visceral Surgery, Hospital of the Goethe University, 60590 Frankfurt am Main, Germany; ^3^German Institute for Cell and Tissue Replacement gGmbH (DIZG), 12555 Berlin, Germany; ^4^Institute of Transfusion Medicine and Immunohematology, Hospital of the Goethe University and German Red Cross Blood Service Baden-Württemberg-Hesse, 60528 Frankfurt am Main, Germany

## Abstract

Bone marrow mononuclear cells (BMCs) are suitable for bone tissue engineering. Comparative data regarding the needs of BMC for the adhesion on biomaterials and biocompatibility to various biomaterials are lacking to a large extent. Therefore, we evaluated whether a surface coating would enhance BMC adhesion and analyze the biocompatibility of three different kinds of biomaterials. BMCs were purified from human bone marrow aspirate samples. Beta tricalcium phosphate (*β*-TCP, without coating or coated with fibronectin or human plasma), demineralized bone matrix (DBM), and bovine cancellous bone (BS) were assessed. Seeding efficacy on *β*-TCP was 95% regardless of the surface coating. BMC demonstrated a significantly increased initial adhesion on DBM and *β*-TCP compared to BS. On day 14, metabolic activity was significantly increased in BMC seeded on DBM in comparison to BMC seeded on BS. Likewise increased VEGF-synthesis was observed on day 2 in BMC seeded on DBM when compared to BMC seeded on BS. The seeding efficacy of BMC on uncoated biomaterials is generally high although there are differences between these biomaterials. Beta-TCP and DBM were similar and both superior to BS, suggesting either as suitable materials for spatial restriction of BMC used for regenerative medicine purposes *in vivo*.

## 1. Introduction

Large bone defects occur frequently in the course of trauma and osteomyelitis or due to tumor resection. The gold standard for the treatment of large bone defects is transplantation of autologous bone grafts, preferably taken from the iliac crest. However, the bone volume at the donor site is limited and the surgical procedure for bone extraction is often accompanied by donor site morbidities such as long-lasting pain [1–3].

The use of osteoinductive cells combined with an osteoconductive scaffold has been proposed as an alternative for the treatment of large bone defects. It has been demonstrated in a variety of animal bone defect models that marrow stromal cells (MSC) combined with endothelial progenitor cells (EPC) increased significantly the bone healing process [4–6]. Our own previous work revealed a significant increase of early vascularization mediated by EPC [7] which foster the bone mass increase supported by MSC in critical size defects of the femur [8, 9] and of the skull [10] in rats.

Despite their beneficial effects on bone healing, the use of culture expanded cells comes with inherent disadvantages, including regulatory ones. To obtain a sufficient number of cells for clinical use, MSC will require several weeks of expansion in culture, markedly delaying definitive surgical repair of the bone defect. There is some evidence that, during that process, MSC may accumulate genetic alterations, which in turn might increase the risk of cancer [11]. Also, some of the growth factors that are used for EPC differentiation* in vitro* such as IGF-1 might support transformation of hematopoietic progenitors [12] from which EPCs develop [13].

Bone marrow mononuclear cells (BMCs) might be a promising alternative to cultured cells, if preliminary data about their osteoinductive properties can be confirmed in humans, specifically also in humans with pathological bone structure. BMCs are a heterogenous mixture of diverse cell types including (immature) lymphocytes, (immature) monocytes, and progenitor cell populations. A BMC preparation evidentially comprises several subsets of regenerative potential such as (immature) monocytes and hematopoietic stem cells (HSCs), a putative source of EPCs, and precursors of MSCs [14–16]. Putative MSC precursors can be identified by the expression of the nervous growth factor receptor-1 (CD271) and the absence of the pan leukocyte marker CD45 [17], whereas EPC can develop from CD34/CD133/CD45 expressing cells [13]. MSC-precursor is a rare population of cells residing in the bone marrow that were defined by the presence of CD271 expression and, respectively, low or absence of pan leukocyte antigen CD45 expression. Those cells were frequently found in close vicinity to CD34+ progenitor cells [17] and possess the potential for trilineage differentiation (adipogenic, chondrogenic, and osteogenic potential) [18]. It has been further shown that the CFU-F concentration correlates well with the concentration of those cells within the bone marrow [19] and that approximately 5% of those cells were capable of forming CFU-F [17, 19].

It has been demonstrated that BMC exerts therapeutic effects by improvement of vascularization as exemplarily demonstrated by Jeon et al. [20] using the hindlimb ischemia model of the mouse. Transplantation of BMC resulted in significantly increased microvessel density [20]. There is further evidence that BMCs also support bone healing. Concentrated, autologous bone marrow aspirate was injected percutaneously into noninfected, atrophic nonunions of the tibia. A positive correlation of the mineralized callus volume with the concentrations of progenitor cells within the injected cell preparation was observed [21]. However, there are hints that red blood cell contaminations, which are common in bone marrow concentrates, might impair the efficacy of autologous bone marrow cell therapy [22].

Oftentimes, in order to spatially restrict regenerative cells, they will be seeded on a carrier before being placed into the bone defect. Different kinds of scaffolds are available which vary in their chemical composition, shape, and surface characteristics. Osteoconductivity, osteoinductivity, and adherence of cells are dependent on material properties and prior work of our group demonstrated significant differences between scaffold types with regard to adherence and metabolic activity of MSC and EPC [23, 24].

Although BMCs probably constitute a feasible and functional alternative to cell-culture based bone tissue engineering applications there is a substantial dearth of information regarding the needs of BMC for adherence and survival on scaffolds suitable for bone tissue engineering. Hence, this work was undertaken to elucidate the role of different surface coatings for the primary adherence of BMC to a *β*-TCP scaffold and, based on those results, to compare the adherence and function of BMC across three different types of scaffolds.

## 2. Materials and Methods

### 2.1. Ethics

Human BMCs were isolated from bone marrow aspirates obtained from the iliac crest. Bone marrow samples were kindly provided by the German Red Cross Blood Donor Service Baden-Württemberg-Hesse, Frankfurt, Germany. The use of anonymous bone marrow samples for research purposes was approved by the local ethics committee (Ethik-Kommission des Fachbereichs Medizin der Johann Wolfgang Goethe-Universität, Project number 329/10) and informed consent was acquired from all donors.

### 2.2. Isolation of BMC

Bone marrow mononuclear cells were isolated according to Assmus et al. [25]. Briefly, bone marrow aspirates were diluted with phosphate-buffered saline (PBS, 1 : 3) and mononuclear cells were isolated by density gradient centrifugation with Ficoll (1.077 g/cm^3^, Biochrom, Berlin, Germany) at 800 g for 20 min without brake at room temperature. Mononuclear cells were washed twice with 25 mL phosphate-buffered saline (800 g) and counted. Viability was assessed by exclusion dye stain using trypan blue.

### 2.3. Biomaterial Characterisation

The scaffolds being tested were the synthetic *β*-tricalcium phosphate (*β*-TCP) Chronos (Synthes, Dübendorf, Switzerland), bovine cancellous bone hydroxyapatite ceramic (BS, Cerabone,* aap Biomaterials*) and human demineralized bone matrix (DBM, DIZG, Berlin, Germany). The *β*-TCP particles have a size of 1.4–2.8 mm, a porosity of 60%, and a pore size of 100–500 *μ*m. This material is further characterized by low mechanical stability and moderate biodegradability.

The BS ceramic has a particle size of 0.5–1.6 mm, a porosity of 65–80%, a pore size of 100–1500 *μ*m, high mechanical stability, and slow biodegradability (data published by the manufacturer).

Demineralized bone matrix (DBM) particles are obtained from human cortical bone and vary between 1 and 3 mm in size. Their porosity equals natural human bone-micropores with a diameter of 1-2 *μ*m that were frequently seen. The DBM particles are sterilized by a validated peracetic acid-based process (data published by the manufacturer).

### 2.4. Coating of *β*-TCP Granules with Fibronectin and Human Plasma

One mL of sterile *β*-TCP, BS, and DBM were incubated in each 1 mL fibronectin solution (10 *μ*g/mL, Sigma, Deisenhofen, Germany) in PBS without Mg^2+^ and Ca^2+^ (PBS^−/−^) or in 1 mL of thawed fresh frozen plasma (German Red Cross Blood Service Baden-Württemberg-Hesse) for 30 min. The supernatants were removed and the granules were air-dried under sterile conditions at room temperature and used within 24 h.

### 2.5. Seeding of BMC and Evaluation of Seeding Efficacy

Scaffolds were placed in a dense monolayer in individual wells (area = 2 cm^2^) of a 24-well plate (Nunc, Wiesbaden, Germany) using sterile forceps. A number of 1 × 10^6^ BMC in a volume of 350 *μ*L was dripped carefully on each scaffold and incubated for 10 min at 37°C. After incubation, medium containing nonadherent cells was removed and dripped once again over the biomaterials, followed by incubation as indicated above. This procedure was repeated three times. The scaffolds were then gently transferred to another well containing 500 *μ*L* MesenCult* medium. The remaining cells in the supernatant and at the bottom of the initial seeding well were isolated. Adhering cells were harvested by a 5 min incubation with Accutase (PAA-Laboratories, Linz, Austria). The cells were counted and the percentage of adherent cells was calculated: ((initial  cell  number − remaining  cell  number)/initial  cell  number)∗100.

### 2.6. Scaffold Surface Characteristics and Direct Proof of BMC by Means of SEM

Surface topography, roughness, and morphology of the biomaterials and adherent BMC were assessed by scanning electron microscopy (SEM). Two days after BMC seeding, untreated and treated scaffolds were fixed with glutardialdehyde (2%) for 30 min and subsequently dehydrated through ascending grades of alcohol (25%, 50%, 75%, 96%, and 100% ethanol) for 15 minutes each step. Scaffolds were then incubated overnight in 1,1,1,3,3,3-hexamethyldisilazane (Merck-Schuchardt, Hohenbrunn, Germany) and drained. Afterwards the samples were sputtered with gold (3 × 60 s, Agar Sputter Coater, Agar Scientific Ltd., UK) and analyzed using a Hitachi FE-SEM S4500 (Hitachi, Dusseldorf, Germany) with a voltage of 5 kV. The images were digitally recorded using the Digital Image Processing System 2.6 (Point Electronic, Halle, Germany).

### 2.7. Characterization of BMC and Determination of Accumulation/Depletion of Progenitor Cells on the Scaffolds

Flow cytometry was applied to determine the frequency of immature hematopoietic stem cells (CD34+/CD133+/CD45+), more mature progenitor cells (CD34+/CD133−/CD45+), and putative MSC-precursors (CD271+/CD45−) in the BMC preparations prior to the seeding procedure as well as in the supernatant after the seeding procedure to determine whether these cell types differ in their adhesive capacity to the scaffolds. Increased adhesion to the scaffold would result in a decreased percentage of that progenitor cell species in the fraction of nonadhering cells and a decreased adhesion to the scaffold would cause a relative increase of that progenitor cell species in the fraction of the nonadhering cells.

5 × 10^5^ BMC were suspended in 100 *μ*L PBS and were incubated for 30 minutes at 4°C with 10 *μ*L of each CD34-FITC (BD-Biosciences, Heidelberg, Germany), CD133-PE (Miltenyi-Biotech, Bergisch-Gladbach, Germany), CD45-PerCP-Cy5 (BD-Biosciences), and CD271-APC (RnD-Systems, Wiesbaden, Germany). Fluorochrome conjugated isotype identical antibodies served as control. After incubation, the cells were washed twice and immediately analyzed by flow cytometry. 1 × 10^5^ mononuclear cells were acquired based on their forward and side scatter properties.

### 2.8. Counting of Adherent BMC

BMCs were seeded on scaffolds as described in the previous chapter. Approximately 4-5 granules were transferred to a single well of a 96-well plate using sterile forceps and incubated in 200 *μ*L* MesenCult* for a period of 2, 7, 14, or 21 days in a CO_2_ incubator at 37°C. At each time point, granules were fixed with 2% paraformaldehyde in PBS^+/+^ for 20 min and subsequently washed gently with 2 × 200 *μ*L PBS per well and followed by further incubation with 1 *μ*L DAPI (2-[4-Amidinophenyl]-6-indolecarbamidine, Sigma-Aldrich, Deisenhofen, Germany) with a final concentration of 1 *μ*g/mL for 10 minutes at room temperature. After the staining, the granules were washed thrice with PBS^−/−^ and transferred to a new well in order to prevent false positive results caused by the adherent cells at the bottom of the cultivation well. Finally, the granules were analyzed by fluorescence microscopy (Zeiss,* Axioobserver*, Gottingen, Germany) and photographed. EPC and DAPI stained MSC were counted on 3–5 randomly chosen scaffold granules at a power of 100-fold. Counting was performed by an independent observer blinded to the group setup. The number of cells was corrected to the area of the well covered by the granules.

### 2.9. Determination of Metabolic Activity

To determine the metabolic activity of scaffold-adherent cells, a* cell proliferation kit I *(*MTT*, Roche Diagnostics, Mannheim, Germany) was performed according to the manufacturer's instructions. MTT assay principle is based on the cleavage of the yellow tetrazolium salt MTT (3-[4,5-dimethylthiazol-2-yl]-2,5-diphenyltetrazoliumbromide) to purple formazan crystals by metabolically active cells. All experiments were performed in duplicate. Briefly, cells were seeded on the scaffolds, as described above and granules were transferred into a 96-well plate (Nunc, Roskilde, Denmark) and cultivated for 2, 7, 14, and 21 days. Prior to addition of the MTT-reagent, granules were transferred to a new 96-well plate in order to prevent false positive results caused by the adhered cells at the bottom of the well. To 90 *μ*L of medium, 10 *μ*L of MTT labelling reagent was added to each well and cells were incubated for 4 h followed by further incubation overnight with a solubilization solution. The supernatant was collected and again transferred to a new 96-well plate and measured absorbance at 570 nm using an ELISA reader (*Infinite M200*, Tecan, Mainz, Germany). As controls, increasing numbers (1000, 2500, 5000, and 10000) of BMC were seeded directly in 96-well plates and assessed separately.

### 2.10. Analysis of Absorption Capacity

Biomaterials (100 *μ*g) were placed in a dense monolayer in 48-well plate and the dry weight was recorded using high-accuracy scales (Sartorius, Gottingen, Germany). 200 *μ*L of water was dripped onto the granules and incubated for 1 min. Nonabsorbed water was removed and the wet weight was determined. The ratio of wet weight to dry weight was calculated. This experiment was repeated 10 times with each of the biomaterials.

### 2.11. Analysis of BMC within DBM Granules

BMC within DBM granules were confirmed by histology. DBM seeded with BMC was embedded in paraffin and 3 *μ*m sections were cut. Sections were deparaffinized with xylene and rehydrated through a series of descending grades of alcohol. The section was stained subsequently either with hematoxylin/eosin (HE) or with DAPI and subjected to microscopy.

### 2.12. Determination of VEGF Release

VEGF levels in the supernatant on 2, 7, 14, and 21 days were measured by ELISA (RnD-Systems, Wiesbaden, Germany) according to the manufacturer's instructions. The medium was replaced 24 h prior to harvest of the supernatants; hence both factors were allowed to accumulate in the medium for 24 h. Medium incubated 24 h from a cell-free scaffold served as control.

### 2.13. Statistics

Results were presented as box plots of the median in diagrams, as median, and as interquartile ranges (median/25% quartile/75% quartile) in the description of the results. A Kruskal-Wallis test with Dunn's post hoc test for multiplicity was used for comparisons between the groups and for the analysis of changes during the follow-up period (day 2 versus day 7 versus day 14 versus day 21). A *P* value < 0.05 indicates statistical significance.

## 3. Results

### 3.1. Characterization of BMC

In order to evaluate the fractions of immature cells with putative regenerative capacities we analyzed the distribution of CD34 positive stem/progenitor cells (CD34+/CD45+), hematopoietic stem cells (CD34+/CD133+/CD45+), and putative MSC precursors (CD271+/CD45−) in BMC preparations obtained from 10 individual donors. The total content of progenitor cells (CD45+/CD34+) within the BMC preparations was 3.64% (1.43%/3.69%), and the percentage of the CD34+/CD133+ subtypes was 0.83% (0.23%/2.04%). The fraction of CD271+/CD45− cells was 0.06% (0.04%/0.07%); that is, all frequencies were within the expected range.

### 3.2. Seeding Efficacy, Adherence, and Metabolic Activity of BMC Depending on Culture Conditions

**Figure 1:**
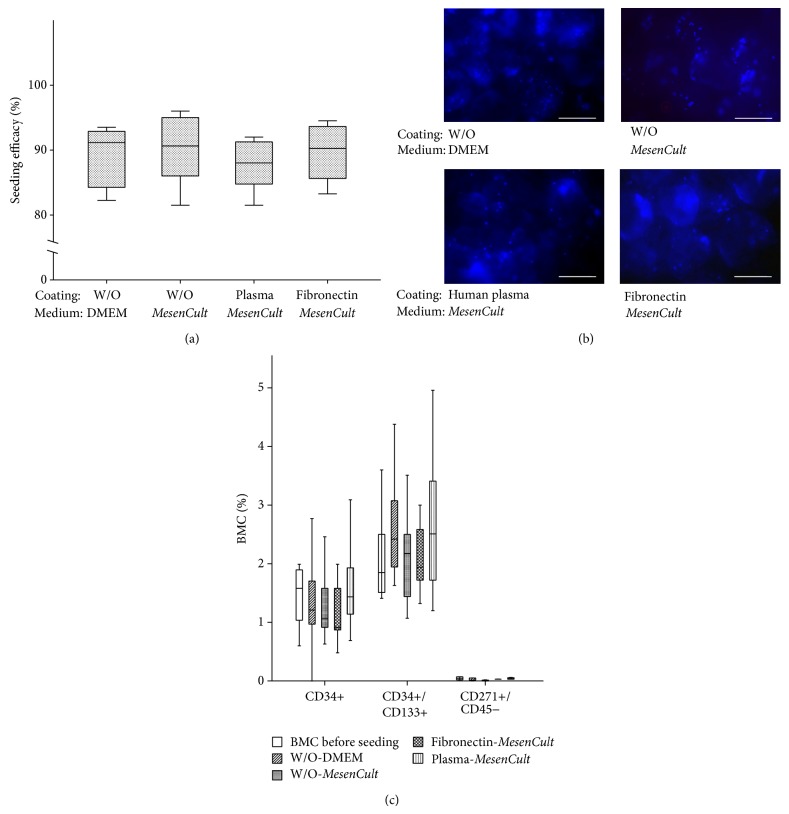
Seeding efficacy of BMC depending on the surface coating and medium conditions. BMCs (*n* = 16) were seeded on *β*-TCP either coated with fibronectin or human fresh frozen plasma and on uncoated *β*-TCP in the presence of* MesenCult* medium or DMEM. Seeding efficacy (%) is plotted on the ordinate. No significant differences in seeding efficacy were observed (a). Representative fluorescence microscopy images of DAPI stained BMC seeded under the different conditions are shown in (b). Similar amounts of adhering BMC were seen under each condition. No selective enrichment or depletion of specific progenitor cell subpopulation was observed on the surface of all biomaterials in various medium conditions as shown in (c). The percentages of progenitor cell types which resemble EPC (CD34+/CD45+; CD34+/CD133+/CD45+) and MSC (CD45−/CD271+) were analyzed in the fraction of nonadhering cells. The similar values indicate that no selective enrichment of progenitor cell subpopulations occurred due to the different culture and surface conditions.

The seeding efficacy was generally high with no significant differences between groups. Neither fibronectin nor human plasma coating improved the adhesion of BMC to *β*-TCP in comparison to uncoated *β*-TCP. Since our clinical product consists of BMC in autologous serum, we also analyzed the adhesion of BMC that was cultured in DMEM medium without serum to evaluate the effect of the serum component on BMC. Adhesion of BMC cultured in serum-free medium and serum-containing medium did not differ significantly (Figures 1(a) and 1(b)). Pertaining to the selective enrichment or depletion of progenitor cells in the supernatant, the progenitor cell concentration in the initial BMC preparation and in the fraction of BMC that did not adhere to the biomaterials was evaluated by flow cytometry. No significant differences were observed (Figure 1(c)). This result indicates that neither surface coating nor medium conditions induced a selective enrichment or depletion of progenitor cell species on the *β*-TCP granules (Figure 1(c)).

The seeding efficacy of BMC on BS and DBM based on the surface coating was also tested in an additional experiment. A fibronectin coating improved the adhesion of BMC to BS significantly in comparison to uncoated BS whereas a precoating with human plasma led to a significant reduction of the seeding efficacy in comparison to uncoated and fibronectin coated BS. Moreover, the generally high retention of BMC to DBM did not yield a better result by surface coatings. It has to be noted that coating with human plasma did not impair the adhesion of BMC on DBM (Table 1).

**Figure 2:**
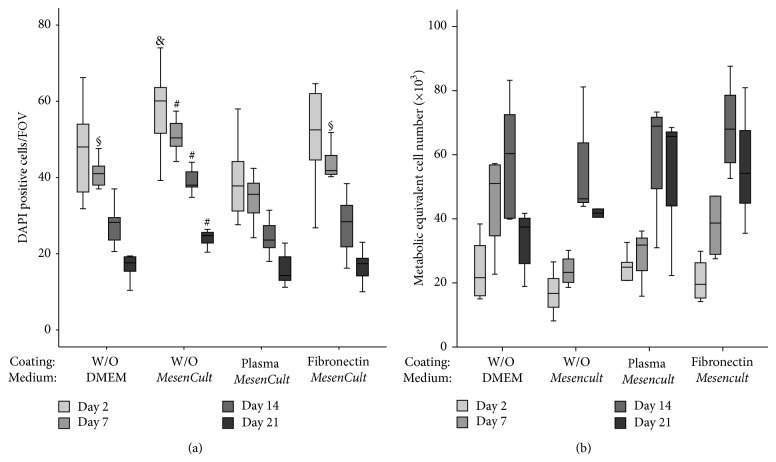
Number and metabolic activity of adhering BMC over the time depending on surface coating and medium conditions. BMCs seeded on *β*-TCP were cultured over 21 days. (a) Number of adhering cells was determined at day 2 (white bars), day 7 (light grey bars), day 14 (dark grey bars), and day 21 (black bars) by means of DAPI staining and subsequent fluorescence microscopy as indicated in Materials and Methods section. Significantly highest numbers of adhering BMC were observed on *β*-TCP without coating and cultured in* MesenCult* medium (*n* = 12). ^§^ *P* < 0.05 without coating in DMEM and fibronectin coating in* MesenCult*, day 7 versus human plasma in* MesenCult*. ^&^ *P* < 0.05 without coating in* MesenCult*, day 2 versus human plasma in* MesenCult*. ^#^ *P* < 0.05 without coating in* MesenCult*, day 7, day 14, and day 21 versus all other groups. The changes of the metabolic activity over 21 days based on a function of surface coating and on medium conditions were presented in (b). The metabolic activity of BMC increased on all biomaterials in all experimental conditions but no statistically significant differences were found (*n* = 7).

The adhesion of BMC on the differently pretreated *β*-TCP was monitored over a period of 21 days by means of fluorescence microscopy and MTT assay. The number of DAPI stained cells declined significantly regardless of the culture conditions. Highest cell numbers were observed on uncoated *β*-TCP in the presence of* MesenCult* medium (Figure 2(a)). Interestingly, the metabolic activity of BMC increased significantly over a period of time in all groups. No significant differences were observed in metabolic activity between the groups (Figure 2(b)). Based on these findings uncoated biomaterials were used for further analysis.

### 3.3. Seeding Efficacy, Adherence, and Metabolic Activity of BMC on Various Biomaterials

**Figure 3:**
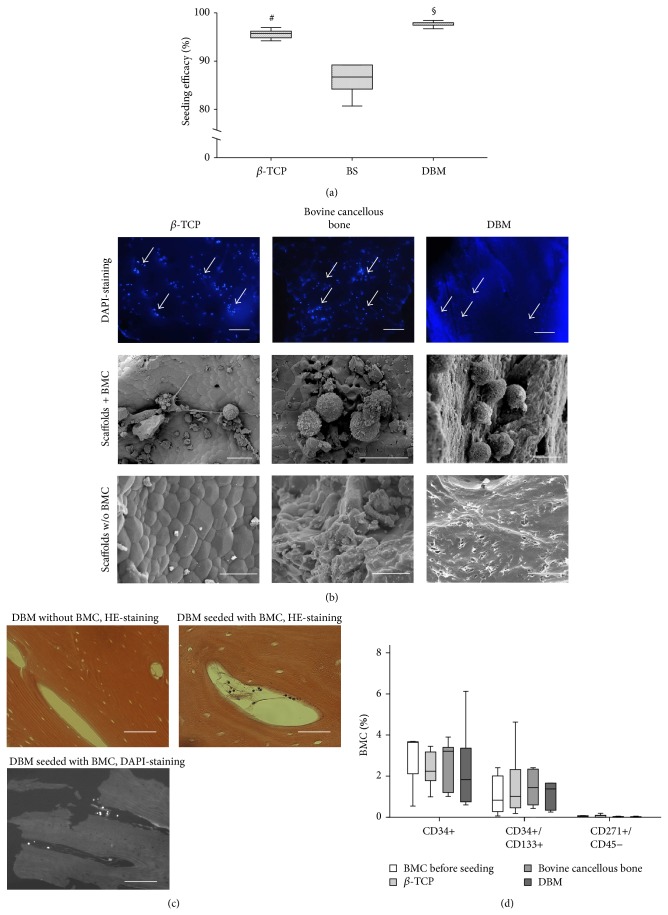
Impaired adhesion of BMC on bovine cancellous bone. (a) BMCs were seeded on uncoated *β*-TCP, DBM, and bovine cancellous bone (BS) and seeding efficacy was assessed by DAPI staining as described in Materials and Methods section (*n* = 7). ^§^ *P* < 0.05, DBM versus *β*-TCP, bovine cancellous bone; ^#^ *P* < 0.05  *β*-TCP versus bovine cancellous bone. Direct confirmation of the presence of BMC on the biomaterials is presented in (b). Upper row column shows DAPI stained BMC on the biomaterials; the middle row shows BMC on the materials' surface by SEM imaging. Cell free biomaterials were shown for comparison in the lower row. (c) BMCs were not only located on the surface of DBM but also found frequently located within the DBM as demonstrated by means of histology. (d) The percentages of progenitor cell types which resemble EPC (CD34+/CD45+; CD34+/CD133+/CD45+) and MSC (CD45−/CD271+) in the fraction of nonadhering cells were presented. No selective enrichment or depletion of progenitor cell subpopulation depending on the biomaterial were noted (*n* = 5). Size bars (b): 200 *μ*m (DAPI-staining), 6 *μ*m (SEM images, middle row), and 10 *μ*m (SEM images lower row); space bars (c): 200 *μ*m (up left), 400 *μ*m (low left), and 50 *μ*m (up right).

A marked lower initial adherence was observed on bovine cancellous bone in comparison to *β*-TCP and DBM, whereas adhesion on *β*-TCP and DBM was at a comparable, high level (Figure 3(a)). Scanning electron microscopy as well as fluorescence microscopy confirmed the adherence of BMC on all biomaterials (Figure 3(b)). Generally, BMC demonstrated a spherical phenotype and often accumulated in clusters. It has to be emphasized that BMCs were frequently found in close vicinity to pore-like structures, when seeded on DBM (Figure 3(b), right panel). Further histological analysis demonstrated that BMCs were also located in channel-like structures within the DBM (Figure 3(c)). The capacity of the biomaterials to absorb liquids may influence the initial adhesion of cells, because cells might be drawn into the biomaterial. Corresponding to the seeding efficacy of BMC we observed a significantly increased absorption of fluid in DBM (111%/113%/109% of dry weight) in comparison to *β*-TCP (98%/99%/97% of dry weight) and bovine cancellous bone (91%/94%/90% of dry weight). The values for *β*-TCP were also significantly higher in comparison to bovine cancellous bone.

Adherent BMCs were detectable on all biomaterials over a period of three weeks. Quantitative results based on counting of DAPI-stained cells are not presented here since the high fluorescence background of DBM hindered the cell counting.

**Figure 4:**
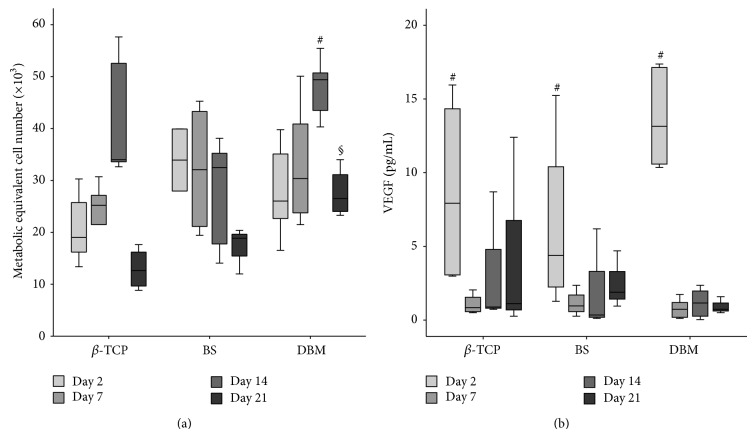
Metabolic activity and VEGF release of BMC depending on biomaterials over a 21-day observation period. (a) Metabolic activity was measured by MTT-assay as described in Materials and Methods (*n* = 7). ^#^ *P* < 0.05 DBM day 14 versus bovine cancellous bone (BS); ^§^ *P* < 0.05 DBM day 21 versus *β*-TCP and bovine cancellous bone. The release of VEGF to the medium is shown in (b). VEGF in the supernatant was assessed by ELISA as described in Materials and Methods section. The VEGF secretion dropped significantly from day 2 to day 21. A trend to higher values on day 2 was seen in BMC cultured on DBM in comparison to BMC seeded on bovine cancellous bone (*P* = 0.09, *n* = 7).

The MTT-assay demonstrated BMC metabolic activity on all biomaterials over the entire observation period. The metabolic activity of BMC seeded on *β*-TCP and DBM increased from day 2 to day 14 and then dropped significantly until day 21 whereas the metabolic activity of BMC seeded on bovine cancellous bone dropped continuously from day 2 to day 21. The metabolic activity of BMC on DBM was significantly increased in comparison to BMC cultured on bovine cancellous bone on day 14. On day 21, the metabolic activity of BMC on DBM was significantly higher than that of BMC seeded on *β*-TCP or bovine cancellous bone (Figure 4(a)).

Next, we analyzed the production of the proangiogenic factor VEGF. VEGF was allowed to accumulate over 24 h in the supernatant before sampling. We observed a generally low VEGF secretion that declined significantly from day 2 to day 21 (Figure 4(b)).

## 4. Discussion

Evidence has provided that bone marrow derived mononuclear cells represent an effective cell source for bone tissue engineering and thus might provide an alternative to the use of long-term cultured cells such as MSC. It is generally accepted that a suitable scaffold is required to bring the cells into and to retain them in the defect site and provide mechanical stabilization of the fracture. So far, protocols and comparisons regarding the seeding and function of BMC on different scaffolds are inadequate. Hence, we evaluated the adherence capacity of BMC on three commercially available scaffolds with or without surface coating and analyzed the adhesion capacity and function of adherent BMC.

We observed that surface coating with either fibronectin or human plasma does not improve the initial adherence of freshly isolated BMC to *β*-TCP scaffolds. The analysis of BMC seeding efficacy and metabolic activity on three different biomaterials revealed significant differences in scaffold-specific biocompatibility.

### 4.1. Effect of BMC in Bone Regeneration

The first use of autologous BMC in bone healing was reported by Hisatome et al. in the year 2005 [26]. They examined “whether the transplantation of autologous BMC can augment neovascularization and bone regeneration in femoral bone defects of rabbits.” BMCs were seeded together with bFGF containing microspheres in a collagen gel and placed into a bone defect in the rabbit medial condyle. A significantly higher vascularization was observed two weeks after implantation and regeneration was significantly improved after eight weeks [26]. Sun et al. (2009) analyzed the effect of BMC on vascularisation and bone formation using a steroid-induced osteonecrosis model of the femoral head in rabbits. As in the report by Hisatome et al. [26], BMC transplanted to the defect zone resulted in significantly higher neovascularization and augmented bone regeneration after four weeks [27]. Those results underline the feasibility of BMC to promote tissue repair processes. Cells within a BMC preparation holding an evidentially regenerative potential are (immature) monocytes and hematopoietic stem cells (HSCs) as putative source of EPCs and precursors of MSCs [13, 16, 22, 25].

### 4.2. Seeding Efficacy: Role of Surface Coating

It is feasible to assume that an increased adhesion to the scaffold during the seeding procedure would result in an enhanced concentration of BMC in the defect site. Increased numbers of BMC might have positive impact on the bone regeneration. This dose effect of bone marrow cells for bone healing has been demonstrated by Hernigou et al. in patients suffering from noninfected, atrophic nonunions of the tibia [21]. They observed a positive correlation of the mineralized callus volume with the concentrations of progenitor cells within the injected cell preparation [21]. However, data are limited about the needs of BMC to adhere to biomaterials. Few reports are available in which BMCs were seeded on biomaterials such as allogeneic bone graft [28], extracellular matrix scaffolds [29], gelatin hydrogel, and porous hydroxyapatite scaffold [30] prior to transplantation. But methodically, surveys and evaluations regarding the compatibility of different biomaterials for BMC were lacking to a wide extent.

Although the seeding efficacy of BMC on the test materials (*β*-TCP, BS, and DBM) was already reasonable we analyzed if additional surface coatings using fibronectin or human plasma could further increase the percentage of adherent BMC. It has been described that fibronectin promotes the adhesion of cells with endothelial-like and angiogenic properties [31]. Moreover, a fibronectin coating can be used to accumulate stem cells with angiogenic properties as well as of MSC from cord blood [32]. In contrast to the literature we observed that fibronectin coating did not increase total BMC adherence to *β*-TCP, the same as to the progenitor cells although similar fibronectin concentrations had been used (20 *μ*g/mL [31], 5 *μ*g/mL [32]), nor did precoating of *β*-TCP and BS with fresh frozen human plasma improve the adherence of BMC; albeit evidence is available that plasma components such as fibrin increase the adhesion of osteoblasts and MSC to scaffolds [33, 34]. Furthermore, our time course analysis demonstrated that surface coating did not improve the long-term adhesion of BMC to *β*-TCP. Our results might indicate that the adhesive properties of the uncoated *β*-TCP scaffold used in this study were strong and sufficient to bind the majority of cells. Furthermore, it can be hypothesized that surface modifications with fibronectin and human plasma might slightly impair the adhesive properties of *β*-TCP and BS by saturation of binding sites or neutralization of its surface charge.

### 4.3. Biocompatibility of the Biomaterials for BMC

We observed that the adherence of BMC differed significantly between the analyzed biomaterials. Highest seeding efficacy of BMC was noted on DBM and *β*-TCP whereas significantly lower percentages of adhering BMCs were found on bovine cancellous bone. Metabolic activity increased on *β*-TCP and DBM at least over 14 days whereas a steady drop of metabolic activity was noted already from day 2 for BMC cultured on bovine cancellous bone.

These results are in line with our previous studies in which we analyzed the adherence and activity of MSC and EPC on six different scaffolds including *β*-TCP, bovine cancellous bone, and human cancellous bone. Both types of cells, MSC and EPC, demonstrated a significantly decreased adherence on bovine cancellous bone in comparison to *β*-TCP and processed mineralized human cancellous chips [23, 24]. Those results suggested that the surface properties of human bone scaffolds facilitated the adherence of human cells in comparison to xenogeneic bone materials.

The high biocompatibility of DBM for human MSC was reported by Kasten et al. (2006). They report an improved seeding efficacy and higher osteocalcin expression in comparison to MSC cultured on *β*-TCP and calcium deficient hydroxyapatite [34]. The differences in adhesion and osteoinductive capacity can be explained by the fact that DBM consists of collagen and contains a variety of adhered growth factors such as bone morphogenetic proteins (BMP) or transforming growth factor- (TGF-) *β* [34]. The presence of the growth factors BMP-2, TGF-*β*1, IGF-1, FGFa, and PDGF in variable concentrations in three different commercially available DBM preparations was also demonstrated by Wildemann et al. [35]. Furthermore, Pietrzak et al. detected BMP-2, -4, and -7 in DBM [36], though it must be stated that specific methods used for sterilization of the DBM can influence the attachment and function of cells as it has been described for osteoblasts [37]. It is safe to assume that the impairment of adhesion and function is not limited to osteoblasts; other cell types may also be affected. The DBM used in this study received a peracetic acid treatment which is a validated and “a reliable sterilization method” [38, 39] but may alter mechanical properties of the DBM [40]. Biocompatibility, however, appears not to be significantly impaired by this sterilization procedure: Endres et al. cultured bone marrow derived cells on differentially pretreated allogeneic bone transplants and assessed cell vitality and the percentages of immune cell subpopulations four weeks after transplantation. Comparable results were obtained for bone marrow derived cells cultured on untreated allogeneic bone or on peracetic acid sterilized bone whereas gamma-irradiated bone exerted significant adverse effects on the transplanted cells [41].

Comparative studies regarding the cellular behavior on bovine cancellous chips are rare. As mentioned earlier in our previous study [23, 24], we observed a significantly impaired seeding efficacy of MSC and EPC in bovine cancellous bone. In accordance, Naujoks et al. (2010) compared the seeding efficacy and the proliferation of human unrestricted somatic stem cells seeded on bovine cancellous bone-based biomaterials in comparison to different *β*-TCP scaffolds. Cells seeded on bovine cancellous bone demonstrated a much lower seeding efficacy and proliferative activity in comparison to cells seeded on *β*-TCP [42]. It is worth to be noted that different bovine materials were analyzed by Naujoks' group and in our studies. The reasons for the comparatively low performance of cells seeded on bovine cancellous bone remain speculative. It is established that the “biocompatibility of biomaterials is influenced by the three dimensional topography and physico-chemical properties of the material surface” [43]. Hence, it can be assumed that a combination of those factors may be responsible for the impaired biocompatibility.

### 4.4. Progenitor Cell Subtype Specific Adhesion on the Biomaterials

Highly adherent and under homeostatic conditions stationary cells such as osteoblasts, MSC, or EPC are typically used to analyze biocompatibility of scaffolds for bone tissue engineering. In contrast, BMC consists of multiple, mostly naturally itinerant cell types with differential adhesion properties. Hence, we presumed that results obtained with those cell types cannot be directly translated to BMC. In the present study, we demonstrated by indirect measurement that putative MSC precursors (CD45−; CD271+) and EPC (CD34+/CD133+) adhered to all tested biomaterials with no selective enrichment or depletion related to the scaffold, although absolute numbers of progenitor cell populations might differ due to differences in scaffold specific properties in the seeding efficacy of BMC. This might be relevant since evidence exists that there is a dose effect relationship between the number of MSC-precursors (CFU-F) and the bone healing response [15, 21].

### 4.5. Differentiation of BMC on Biomaterials

To our present knowledge, the differentiation of BMC cultivated on biomaterials has not been addressed yet. BMC has a significant amount of hematopoietic progenitor cells as identified by the phenotype CD34+ CD45+. It has been demonstrated that those cells were capable of endothelial differentiation and support the vascularization of ischemic tissues. Furthermore, monocyte precursors can also be differentiated towards an endothelial-like phenotype [44, 45]. Both cell types were often referred to as endothelial progenitor cells due to their ability to obtain endothelial characteristics. A further subdivision into early EPC (arising from monocytic precursors) and late EPC (arising from HSC) is often made to define the cellular origin of the EPC. However, both types of EPCs need stimuli such as VEGF to acquire an endothelial-like phenotype and endothelial functionality [13]. Those stimuli were not present in the culture medium that was used in our experiments; hence it is unlikely that a significant endothelial differentiation took place. This assumption can be shown by the low VEGF synthesis observed in our experiments at a later time point, since VEGF release is a key feature of EPCs [45].

The differentiation of BMC cell fractions towards the osteogenic lineage is of great interest for the purpose of bone tissue engineering. As mentioned before, MSC precursor was identified by the expression of CD271 and absence of CD45 [17–19]. Osteogenic stimuli such as BMP-2 are needed to induce the osteogenic differentiation of MSC* in vivo* [46]. Wildemann et al. [35] have shown that demineralized bone matrix contains growth factors such as BMP-2, IGF-1, and FGF that facilitate the proliferation and osteogenic differentiation of MSC; hence, it has been described that DBM exerted a significant osteoinductive effect on MSC* in vitro* [47, 48]. Likewise *β*-TCP also has osteoinductive properties on MSC* in vitro*. The cultivation of human bone marrow MSC on *β*-TCP-scaffolds increased alkaline phosphatase-activity as well as the expression of osteogenesis related genes such as runx2, alkaline phosphatase, osteopontin, and osteocalcin [49]. Although some of the biomaterials have significant osteoinductive properties on MSC, the low concentration of MSC in the BMC fraction has to be taken into account. Therefore, it remains elusive whether osteogenic differentiated MSC within transplanted BMC will exert a significant effect on the bone healing response.

### 4.6. Secretion of Mediators

Our previous work has shown that vascularization is an early event in cell based bone repair approaches [7]. It is hypothesized that the established proangiogenic effect of BMC is mediated at least partially by paracrine effects such as release of VEGF [50]. We therefore analyzed the VEGF secretion by BMC seeded on *β*-TCP, bovine cancellous bone, and human DBM. We observed a significant VEGF release to the medium only on day 2 after seeding. The concentration of secreted VEGF in the supernatant dropped to a negligible amount during the subsequent observation period. Since angiogenic/vasculogenic activity takes place in early event [7], the short period of VEGF release by BMC might be sufficient to support the establishment of a new capillary net.

## 5. Conclusion

The possibility of a cell based therapy for large bone defects gains increasing attention and it has been demonstrated that BMCs lead to bone healing with effects comparable to cultured MSC and EPC but being applicable within 24 h and without the possible safety issues of the latter. However, less attention has paid to the needs of BMC in terms of a suitable carrier material and its surface coating. The current study provides evidence that uncoated *β*-TCP and DBM are ideal scaffolds for BMC-mediated bone therapy. Alternative carrier materials are available; however, none of these materials significantly exceeded the capacity of *β*-TCP and DBM for cell retention. Near-perfect retention for all different assessed stem/progenitor cell populations was observed, which likely indicates nonspecificity of cell-matrix interactions and allows the conclusion that all contributory cells are quantitatively retained by *β*-TCP or DBM. Within the limits of our results, we may assume that BMC seeded on *β*-TCP and DBM will be useful to improve and enhance the bone regeneration process in future clinical studies. However, generating further data with these materials in large bone defect animal models is a first priority.

## Figures and Tables

**Table 1:** Seeding efficacy (%) of BMC on *β*-TCP, BS, and DBM in dependancy on the surface coating. Biomaterials were coated with either fibronectin (10 *μ*g/mL) or human plasma 24 h prior the experiment. *MesenCult* medium was used. Values are presented as median/25% quartile/75% quartile; *n* = 5-6, ^*^ *P* < 0.05.

	No coating	Fibronectin	Plasma
*β*-TCP	87.0/82.3/94.5	87.0/84.3/94.5	86.0/79.3/89.0
BS	81.0/78.5/88.0	93.0/90.3/96.3 ^*^versus no coating ^*^versus plasma	78.5/71.8/80.8
DBM	91.5/89.5/94.3 ^*^versus BS	97.5/86.5/98.5	92.5/90.3/96.3 ^*^versus BS, *β*-TCP

## Abbreviations

BMC: Bone marrow mononuclear cells
BS: Bovine cancellous bone
*β*-TCP: *β*-Tricalcium phosphate
DBM: Demineralized bone matrix
EPC: Endothelial progenitor cells
MSC: Marrow stromal cells
SEM: Scanning electron microscopy
VEGF: Vascular endothelial growth factor.
